# Dual-energy and perfusion CT for predicting response to chemo-radiotherapy in head and neck cancer: an exploratory study

**DOI:** 10.3389/fonc.2026.1762607

**Published:** 2026-05-05

**Authors:** Benjamin Van Honacker, Yolène Lefebvre, Marianne Paesmans, Manuela Burghelea, Dirk Van Gestel, Tatiana Dragan

**Affiliations:** 1 Université libre de Bruxelles, Brussels, Belgium; 2 Institut Jules Bordet, Brussels, Belgium

**Keywords:** chemo-radiotherapy response, dual-energy CT (DECT), head and neck cancer (HNC), perfusion CT (PCT), quantitative parameters

## Abstract

**Introduction:**

Head and neck cancer (HNC) is a major global health issue, often linked to smoking, alcohol, and viruses (HPV, EBV). HPV-driven oropharyngeal squamous cell carcinoma (OPSCC) showed distinct biology and better response to chemoradiotherapy (CRT) than HPV-negative HNC. Despite these differences, treatment remains uniform, emphasizing the need for predictive imaging biomarkers. Dual-energy computed tomography (DECT) and perfusion computed tomography (PCT) are promising techniques for predicting treatment response, though their clinical utility remains underexplored. This exploratory study investigates whether quantitative parameters (QPs) from DECT and PCT, can predict complete response (CR) 12 months after CRT.

**Materials and methods:**

36 HNC patients were enrolled and classified as CR or non-CR (NCR) based on RECIST 1.1 criteria at one year. Imaging was performed at baseline, 3 weeks, 3 months, and 12 months, measuring blood flow (BF), blood volume (BV), and contrast media attenuation (CMA). Statistical analyses compared CR and NCR groups, including subgroups based on HPV status.

**Results:**

Patients with CR (n=26) showed significantly higher BF and BV at 3 weeks (p<0.01), longer mean transit time (MTT) at 3 months (p<0.03), and lower CMA at 3 months (p<0.03) compared to NCR patients. In the HPV-positive subgroup, CR patients had higher BF and BV than NCR patients (p<0.02). Delta analysis revealed no significant differences except for ΔCMA (3W–0), which was lower in HPV-negative patients (p<0.04).

**Conclusion:**

In this exploratory study, DECT and PCT QPs showed potential associations with treatment response in HNC, particularly at early stages. These hypothetical findings warrant further validation in dedicated prospective studies before integration into personalized treatment strategies.

**Clinical Trial Registration:**

https://clinicaltrials.gov/ct2/show/NCT04019548, identifier NCT04019548.

## Introduction

Head and neck cancer (HNC) represents a biologically and clinically heterogeneous group of malignancies, with oropharyngeal squamous cell carcinoma (OPSCC) increasingly driven by human papillomavirus (HPV) infection, particularly HPV-16. HPV-positive OPSCC is characterized by distinct tumor biology and a markedly better response to chemoradiotherapy (CRT) compared with HPV-negative, tobacco-related HNC (1–3). However, despite these prognostic differences, treatment for advanced disease generally remains uniform (4–6), underscoring the need for reliable imaging biomarkers capable of predicting therapeutic response and enabling individualized treatment strategies. Previous studies have shown the potential of functional MRI, dynamic contrast-enhanced CT, and FDG PET-CT in predicting therapeutic responses in HNC) (7–9). However, their limitations include high costs, long scan times, low resolution for small lesions, and complex quantitative analyses. Dual-energy CT (DECT) provides a promising alternative by offering detailed quantitative and qualitative tissue analyses through DECT derived material decomposition (MD) maps. DECT also enhances iodine contrast at low-energy settings, generates iodine maps, and calculates tissue iodine concentrations, expanding its utility in oncology (10). Despite these advantages, its use in assessing and predicting therapeutic outcomes in advanced HNC remains limited and underexplored.

This prospective exploratory study aims to evaluate and compare DECT- and PCT-derived quantitative parameters as potential imaging biomarkers for predicting treatment response in advanced HNC.

## Materials and methods

### Study design and patients

Our study addresses effectiveness of PCT and DECT-derived quantitative parameters (QPs) in the prediction of therapeutic responses, one of the exploratory endpoints of the Swall-PEG trial (11). This prospective, randomized phase III study was set up to define the place of a feeding tube in the treatment of loco-regional advanced HNC, i.e. previously untreated, histologically proven squamous cell carcinoma of the oral cavity, oropharynx, hypopharynx, larynx, or nasopharyngeal carcinoma. Patients are older than 18 years with an Eastern Cooperative Oncology Group (ECOG) performance status ≤ 2 and candidate for concurrent CRT. Local ethics committee approval was obtained before the start of the study, and all patients gave written informed consent (CE 2982, Swall PEG; IJB-RT-HNC-001). Patients are randomized to the treatment arms (experimental arm versus standard arm) in a 1:1 design (NCT04019548) (11). The primary endpoint of the study is the dysphagia score measured by MD Anderson Dysphagia Inventory (MDADI) at 6 months of follow-up. It is important to note that our current imaging analysis represents an exploratory investigation of quantitative imaging parameters collected simultaneously as a secondary endpoint within the Swall-PEG trial.

### Treatment planning

All patient underwent CRT. The primary tumor and pathological lymph nodes were contoured separately and defined as gross tumor volume (GTVs). Macroscopic tumor sites were treated with a total dose of 69.12 Gy in 32 fractions of 2.16 Gy per fraction by simultaneous integrated boost (SIB) volumetric modulated arc RT (VMAT). The elective nodal volumes were delineated according to international guidelines (12, 13) and treated with a dose of 56 Gy. Fractionation scheme, Planning Target Volume (PTV) margins, and quality assurance were conducted according to the institutional guidelines. The concurrent CRT consisted of cisplatin 100 mg/m² intravenous (IV) on Days 1 and 22 or weekly cisplatin 40 mg/m² IV on Days 1, 8, 15, 22, 29, and 36.

### Imaging

**Figure 1:**
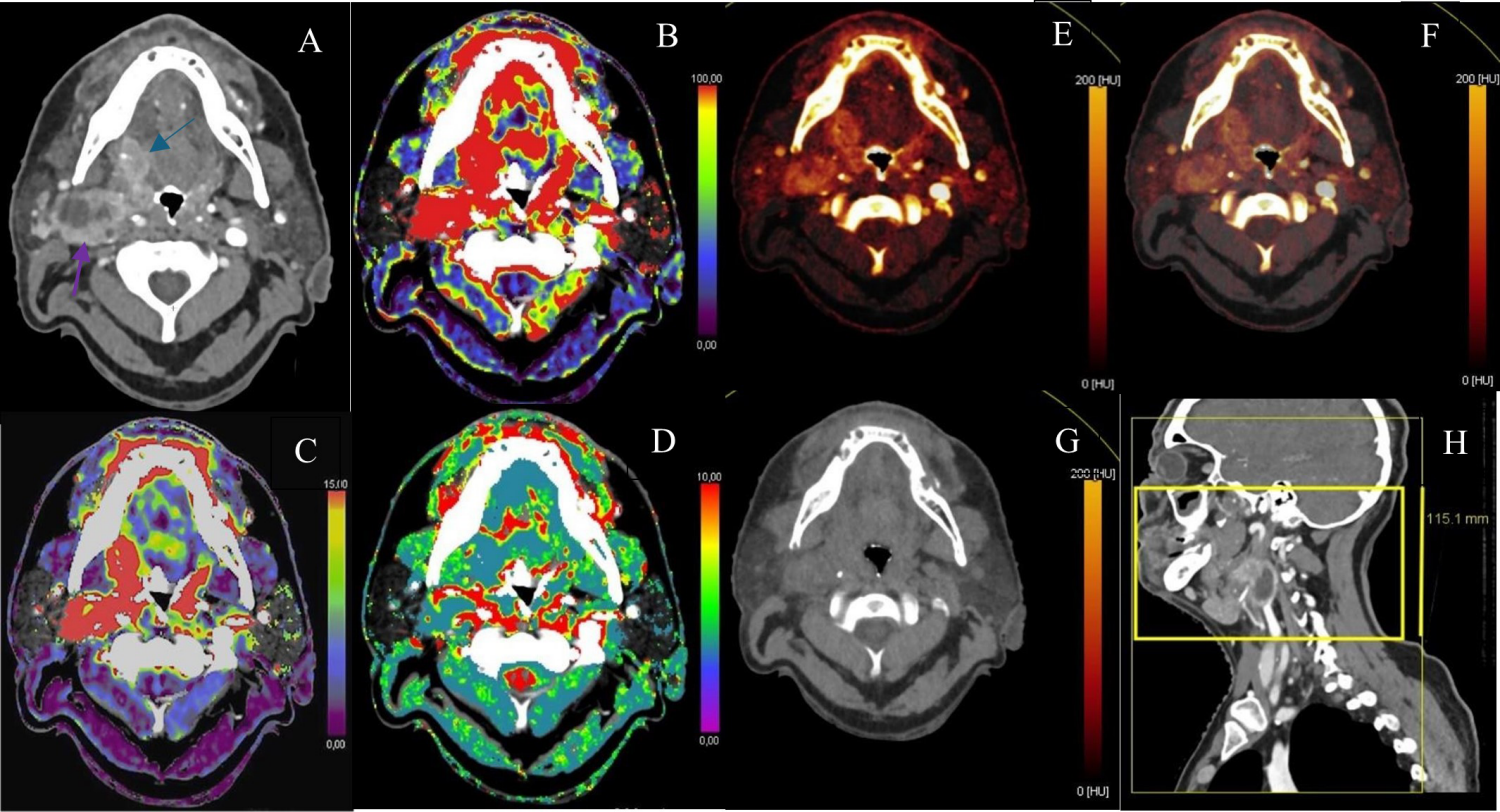
Pre-treatment imaging of a HPV-negative patient with a tonsil and tongue base invading primary tumor (blue arrow) with a partially necrotic right level 2a lymphadenopathy (purple arrow). **(A)** Axial injected DECT. **(B)** Blood flow acquisition by dynamic PCT. **(C)** Blood volume acquisition by dynamic PCT. **(D)** Mean transit time generated by dynamic PCT. **(E)** Iodine map generated by DECT. **(F)** Iodine map generated by DECT: other contrast. **(G)** Virtual non contrast attenuation map generated by DECT. **(H)** acquisition box of iodine map (light yellow delimitation); acquisition box of dynamic PCT (neon yellow delimitation).

The tumor response is measured by DECT (Somatom Force, Siemens Medical Systems, Erlangen, Germany) 3 weeks after the start of CRT, and at 3 and 12 months after the end of treatment. Complete response (CR) is defined as the disappearance of all lesions at the 12 months evaluation, with no correlation being performed with clinical status or PET-CT follow-up. Based on the RECIST 1.1 criteria, each subject was assigned to CR or non-CR (NCR) (14). The latter group gathers the more detailed categories “partial response (PR)”, “stable disease (SD)” and “progressive disease (PD)”. The number of patients per category and per analyzed time period are shown in **Appendix A, Table A**. Examination starts with a PCT focused on both arterial and venous phases of the oropharyngeal tumor. A dynamic “table shuttle” acquisition with tube current modulation is performed at 150 kV, followed a few seconds later by a head and neck (HN) DECT performed on the same scanning device at 80 and 150 kV (Kilovolt), using a bolus tracking method. Biphasic injection protocol is used, typical for HN CT scans. The first phase consists of injecting 60 ml of iomeprol 400 mg/ml at the rate of 4 ml/sec followed by 30 ml of saline solution at the same rate. The second phase starts 33 seconds later with the injection of 40 ml of iomeprol at the usual rate of 3 ml/sec followed by 40 ml of saline solution at the same rate. The analysis of the classical reconstructed HN native CT images acquired during perfusion phase is done with the PACS (Telemis, Louvain-la-Neuve, Belgium). Analysis of the PCT and of the DECT is made with the Syngo software (Siemens Medical Systems). This generates perfusion and iodine maps. During post-processing, the regions of interest (ROI) are placed within the primary tumor area (central slice of the tumor), and in some cases into lymph node cores (see “Results”) by a single 14 years’ experienced radiologist. All QP were automatically calculated within this ROI, blood flow (BF), mean transit time (MTT) and blood volume (BV on the PCT while iodine concentration (IC), virtual unenhanced attenuation (VNC), contrast media attenuation (CMA), fat fraction volume (FFV) and mixed density (MD) on the iodine map generated from the DECT. The definition of these QPs can be found in **Table 1**, and their translation into tomographic images of perfusion or iodine maps are shown in **Figure 1**. Despite having no relevance to tumor perfusion, FFV was a part of this study’s analysis because of its automated calculation by the Syngo software. Also, some studies such as the ones of Hermans et al. (15) and Truong et al. (16), have highlighted λHU as a significant predictor of CR, calculated using the formula λHU = (CT_lower kV – CT_higher kV)/(higher kV – lower kV). However, this quantitative parameter was not included in the study, to avoid overload of the statistical analyses and comparisons, as the number of selected quantitative parameters was already substantial.

**Table 1:** Perfusion and dual-energy computed tomography quantitative parameters definitions (8): PCT, perfusion computed tomography; QP, quantitative parameter; DECT, dual-energy computed tomography; HU, Hounsfield Unit; keV, kiloelectronvolt; ROI, region of interest; QPs, quantitative parameters.

QPs	Name	Definition
PCT QP	Blood flow (BF; mL/min)	Rate of blood flow within the vascular system of a tissue area. Includes flow information from all types of arterial or venous vessels, comprising the arteriovenous shunts which are more common in neoplastic tissue than in healthy ones.
	Blood volume (BV; mL/100mL of tissue)	Blood volume flowing within vascular tissue.
	Mean transit time (MTT; seconds)	Average duration for blood to traverse from the arterial to the venous terminus within the microvascular system. MTT exhibits an inverse relationship with BF.
DECT QP	Iodine concentration (IC; mg/mL)	Amount of iodine (derived of contrast agent) measured in a tissue area.
	Mixed density (MD; HU)	Measurement of the density of the region of interest (ROI) on the virtual monochromatic reconstruction (VMI) at 40 keV.
	Virtual non contrast attenuation (VNC; HU)	Measurement of the attenuation within the ROI on the virtual non contrast reconstruction of iodine map
	Contrast media attenuation (CMA; HU)	Measurement of the attenuation of contrast agent within the ROI on the native CT images.
	Fat fraction volume (FFV; %)	Percentage of fat volume within the ROI

QPs, quantitative parameters.

### Statistical analysis

Quantitative parameters and clinical data were analyzed using R software. Quantitative data distribution was assessed graphically to guide statistical evaluation. Qualitative variables were expressed as frequencies, while quantitative variables, due to asymmetry, were represented by medians and interquartile ranges. Comparisons of quantitative variables between groups were performed using the Wilcoxon test, accounting for abnormal data distribution and small sample sizes. Qualitative variables were compared using Fisher’s exact test. Statistical significance was set at a threshold of α = 0.05. The analysis consisted of comparing the absolute values of QPs between CR and NCR groups, assessing QPs between HPV-positive and HPV-negative oropharyngeal cancers, and subgroup comparisons of QPs within HPV-negative and HPV-positive oropharyngeal cancers. These comparisons were predefined collegially. Data at 3 months for the HPV-negative group were excluded due to limited sample size. A multivariate analysis was also conducted to examine combined predictive factors. In case of CR, QPs could not be assessed due to the tumors becoming undetectable on imaging, explaining the gradual reduction in the number of lesions analyzed at 3 and 12 months post treatment (see **Table 2**). Imaging at the 12-month mark served a focused purpose: determining whether patients achieved a CR or NCR. Although QPs were collected during this time, they were excluded from the primary evaluation due to a lack of data caused by the high number of CR and also to avoid overanalysis. Additional evaluations were launched by adding the comparisons of deltas (noted Δ (t1-0)) between the 3 weeks and baseline values for all QPs (and between 3 months and baseline for BF). No correction for multiple test was applied.

**Table 2:** Number of participants, number of primary tumors and lymph nodes analyzed.

n	Pre-treatment (baseline)	3 weeks post start CRT	3 months post end CRT	12 months post end CRT
Participants (*n*)	35	35*	33	33
Primary tumor (*n*)	30	30	10	7
Lymph nodes (*n*)	34	39	11	3

CRT, chemoradiotherapy. *were included two patients who then died before 3 months follow-up (cf. Figure A in supplementary data).

## Results

36 patients (29 males and 7 females, mean age 63.9 ± 8.5 years; median age 65 +- 10 (interquartile range) years) were recruited between 2020 and 2023. Their characteristics, inclusion and follow-up processes are detailed in **Table 3** and **Appendix A (Figure A).**

**Table 3:** Patients’ characteristics and site, subsite, T, N stage and HPV status of the different tumors.

Site	n	n/subsite				T stage				N stage				HPV status		
						T1	T2	T3	T4	N0	N1	N2	N3	Positive	Negative	No test
Oropharynx	**30**	12	8	6	4	5	17	3	5	2	16	8	4	22	8	0
Subsite		Tonsil	Base of Tongue	Vallecule	Mucosal wall											
Larynx	**2**	1	1			1	1	0	0	0	0	2	0	1	1	0
Subsite		Supraglottic	Vocal cord													
Hypopharynx	**1**	1				1	0	0	0	0	0	1	0	0	0	1
Subsite		Piriform Sinus														
Nasopharynx	**3**					3	0	0	0	0	3	0	0	0	2	1

Bold numbers represent the total number of tumors in each site (which is itself divided into subsites), namely the oropharynx, larynx, hypopharynx, and nasopharynx.

Over the course of the study, the characteristics and number of primary tumors and lymph nodes analyzed were documented at each time point, as outlined in **Table 2**.

**Figure 2:**
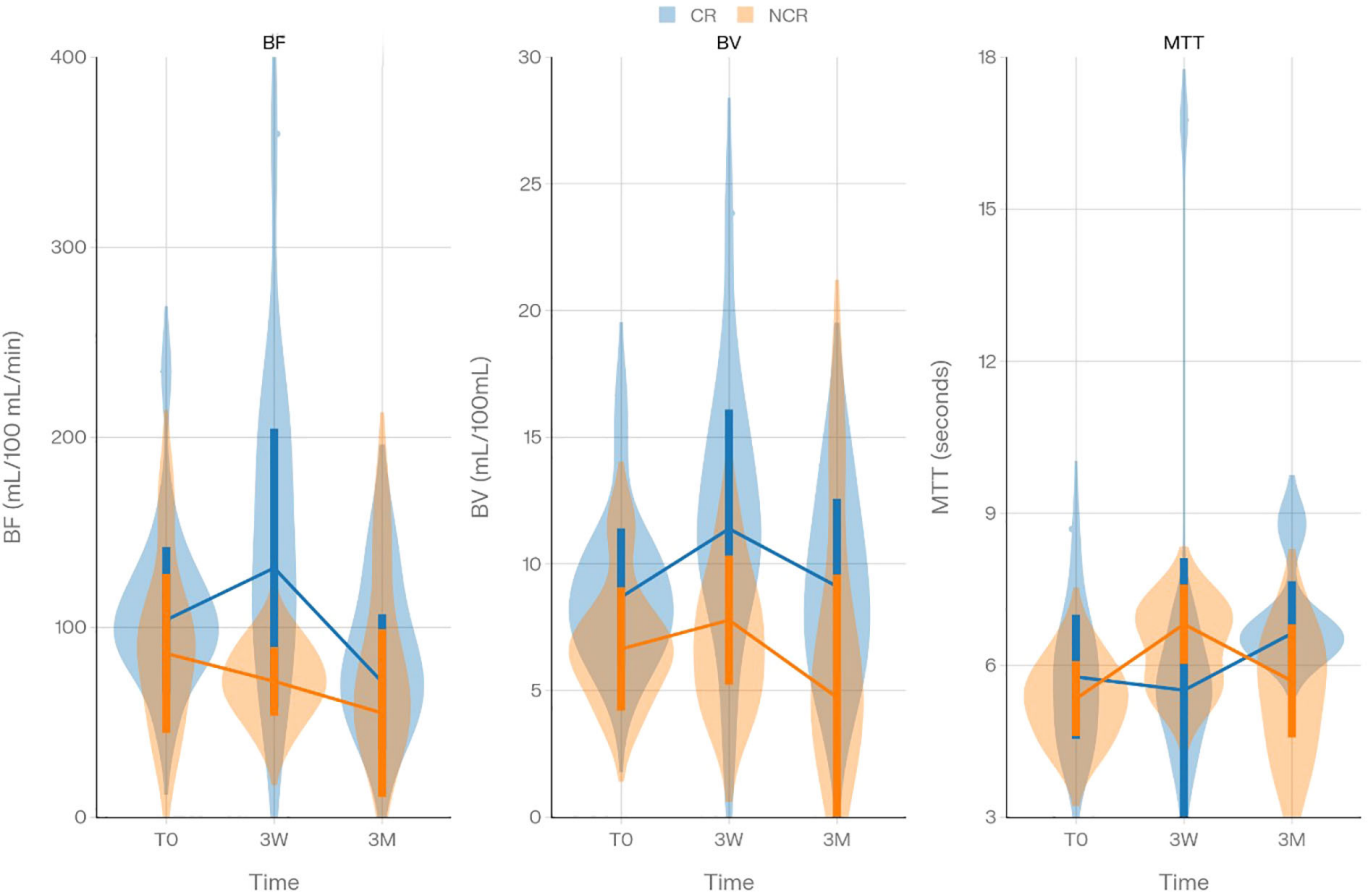
Violin plots showing the distribution of blood flow (BF), blood volume (BV), and mean transit time (MTT) in independent patient samples according to response status (CR vs NCR) at the different assessment time points.

Importantly, all included cases added valuable insights. For example, two patients exhibited malignant lymph nodes without a detectable primary tumor; these cases were included to evaluate lymphadenopathy progression. A high degree of data completeness was maintained, with a few exceptions: one patient missed the PCT modality at 3 months due to a procedural oversight, and another patient developed a secondary lesion at the 3-week follow-up, which was subsequently assessed using DECT and PCT, contributing additional data to the study. The results, detailed in the supplementary tables which can be found in **Appendix A**, reveal several significant findings. BF and BV at 3 weeks (BF_3W, BV_3W) and MTT at 3 months (MTT_3M) were significantly higher in CR subjects compared to NCR ones (p < 0.01, p < 0.01, and p < 0.03, respectively) (**Table 4**). The temporal evolution of BF, BV and MTT is illustrated in **Figure 2**. Conversely, CMA at 3 months (CMA_3M) was notably lower in the CR group (p < 0.032) (**Appendix A, Table C**). In HPV-positive patients, BF_3W and BV_3W were significantly elevated in the CR group (both p < 0.017) (**Appendix A, Table E**). However, no significant differences in QPs were observed between CR and NCR groups among HPV-negative patients (**Appendix A, Table D**). Similarly, the multivariate analysis did not identify significant differences between CR and NCR groups across the study population (**Appendix A, Table B**). The only significant difference in additional analyses was a change in CMA from baseline to 3 weeks (ΔCMA (3W-0)), which distinguished HPV-positive from HPV-negative patients. Full statistical details and additional findings are provided in the supplementary tables for reference.

**Table 4:** Comparison of PCT and DECT QP measured at baseline (0), 3 weeks (3W) and 3 months (3M) post beginning of treatment between CR and NCR (overall patients).

Variable	CR (range) – n = 26			NCR (range) – n = 8*		p-value^2^
	n		Summary^1^	n	Summary^1^	
GTVp	26	9.82 (1.63 - 31.26)		7	9.18 (2.51 - 42.64)	0.32
GTVn	24	12.4 (0.79 - 102.02)		7	19.51 (4.2 - 136.41)	0.1
PCT absolute values						
BF_0	24	104 (83 - 122)		7	86 (70 - 104)	0.4
BF_3WS	22	131 (106 - 190)		7	72 (61 - 82)	**0.004**
BF_3M	5	71 (63 - 97)		5	55 (52 - 86)	0.7
BV_0	24	8.69 (7.13 - 10.37)		7	6.64 (5.91 - 7.45)	0.076
BV_3W	22	11.4 (9.4 -14.5)		7	7.8 (5.0 - 8.1)	**0.006**
BV_3M	5	9.1 (6.2 - 0.4)		5	4.7 (3.8 - 7.1)	0.3
MTT_0	24	5.77 (4.87 - 6.31)		7	5.34 (4.82 - 5.73)	0.6
MTT_3W	22	5.51 (4.98 - 6.12)		7	6.81 (5.61 - 6.98)	0.067
MTT_3M	5	6.63 (6.39 - 6.69)		5	5.69 (4.73 - 6.33)	**0.032**
DECT absolute values						
MD_0	24	117 (103 - 135)		7	117 (109 - 142)	0.6
MD_3W	22	149 (134 - 176)		7	142 (140 - 167)	0.7
MD_3M	5	93 (74 - 107)		5	130 (129 - 164)	0.056
VNC_0	24	40 (35 - 46)		7	42 (34 - 46)	>0.9
VNC_3W	22	34 (27 - 41)		7	33 (32 - 39)	>0.9
VNC_3M	5	30 (14 - 34)		5	22 (21 - 27)	0.5
CMA_0	24	79 (63 - 103)		7	92 (75 - 100)	0.6
CMA_3W	22	122 (96 - 144)		7	108 (104 - 132)	0.7
CMA_3M	5	82 (77 - 93)		5	137 (129 - 145)	**0.032**
IC_0	24	2.40 (2.00 - 3.40)		7	2.80 (2.30 - 3.35)	0.5
-IC_3W	22	3.90 (3.10 - 4.55)		7	3.40 (3.20 - 4.30)	>0.9
IC_3M	5	2.90 (0.60 - 3.60)		5	4.50 (4.30 - 4.60)	0.059
FFV_0	24	12.4 (9.2 - 14.7)		7	9.8 (7.6 - 15.0)	0.8
FFV_3W	22	17 (12 - 23)		7	15 (13 - 19)	0.7
FFV_3M	5	21 (18 - 37)		5	24 (21 - 46)	0.7
PCT Δ (t1-0)						
Δ BF (3W-0)	22	34.12 (11.2 - 73.7)		6	-4.45 (-22.0 - 22.6)	0.055
Δ BF (3M-0)	5	-2.98 (-28.31 - 5.18)		5	-13.94 (-30.01 - 14.81)	0.42
Δ BV (3W-0)	22	3.2 (0.7 - 5.35)		6	1.05 (-1.54 - 3.29)	0.11
Δ MTT (3W-0)	22	-0.19 (-0.8 - 0.9)		6	0.5 (0.17 - 1.36)	0.1
DECT Δ (t1-0)						
Δ MD (3W-0)	22	30.95 (5.6 - 48.5)		6	25.05 (15.7 - 37.6)	0.32
Δ CMA (3W-0)	22	35 (18.4 - 47.7)		6	38.8 (25.4 - 46.4)	0.34
Δ IC (3W-0)	22	1.05 (0.7 - 1.6)		6	1.15 (0.8 - 1.4)	0.42

^1^Median + interquartile range (IQR).

^2^Wilcoxon rank sum exact test- Wilcoxon rank sum test.

CR, complete response; NCR, non-complete response; BF, Blood Flow; BV, Blood Volume; MTT , Mean Transit Time; VNCA, Virtual non contrast attenuation; CMA, Contrast media attenuation; MD, Mixed density; IC, Iodine concentration; FFV, Fat fraction Volume; GTVp, Gross tumor volume, GTVn, Gross nodal volume; Δ BF (0-3W), Difference in Blood Flow between 3 weeks and baseline); Δ BF (0-3M), Difference in Blood Flow between 3 months and baseline; Δ BV (0-3W), Difference in Blood Volume between 3 weeks and baseline; Δ BV (0-3M), Difference in Blood Volume between 3 months and baseline; Δ IC (0-3W), Difference in Iodine Concentration between 3 weeks and baseline; Δ IC (0-3M), Difference in Iodine Concentration between 3 months and baseline; Δ CMA (0-3W), Difference in Contrast Media Attenuation between 3 weeks and baseline; Δ CMA (3M-0), Difference in Contrast Media Attenuation between 3 months and baseline; Δ MD (0-3W), Difference in Mixed Density between 3 weeks and baseline; Δ MD (3M-0), Difference in Mixed Density between 3 months and baseline; Δ MTT (0-3W), Difference in Mean Transit Time between 3 weeks and baseline; Δ MTT (3M-0), Difference in Mean Transit Time between 3 months and baseline. Bold values represent the significative p-values (<0,05).

## Discussion

Evaluation and prediction of response by PCT QPs revealed a trend toward an initial increase in BF and BV median values in the CR group from baseline to the 3-week imaging, followed by a decrease at 3 months. In contrast, the NCR group shows a consistent decrease tendency of BF and a stagnation/slight increase of BV at 3 weeks. These findings are illustrated in **Figure 2**. The nearly significant difference in Δ BF (3W-0) between subgroups (p<0.055) suggests a potential trend that could indicate treatment response. This fluctuation in perfusion parameters can be explained by the effects of RT, which may first induce a transient phase of inflammation and hyperemia. According to Garcia-Figueiras et al. (17), RT can cause endothelial cell damage, leading to increased blood flow and volume within the tumor. This is associated with the release of cytokines such as vascular endothelial growth factor (VEGF), which contributes to tumor hyperperfusion. As treatment progresses and tumor cells die, a reduction in perfusion parameters is typically observed. The absence of the initial increase in BF and BV at 3 weeks in the NCR group may therefore indicate a lack of effective response, potentially predicting treatment failure at 12 months. Baseline BF and BV values were slightly higher in the CR group compared to the NCR group in our study, though these differences were not statistically significant. This finding aligns with previous studies, such as those summarized by Preda et al. (8), which reviewed the role of PCT in monitoring and predicting treatment responses in HNC. Specifically, studies by Hermans et al. (15) and Truong et al. (16) found that higher pre-treatment BF values correlated with better locoregional response to RT. This correlation is supported by the well-established relationship between tumor oxygenation and RT effectiveness. Tumors with higher perfusion are more oxygenated, which allows RT to generate more free radicals, leading to greater tissue damage and an enhanced therapeutic response (18). However, some studies suggest that high perfusion may also be associated with more aggressive tumor behavior. This may be due to hypoxia, which induces radioresistance and stimulates neovascularization, leading to higher perfusion levels (19). Another hypothesis suggests that tumor hypoxia fluctuates depending on systemic arterial pressure, which can therefore vary due to episodes of vasoconstrictions (20). Interestingly, a study by Grkovski et al. (21) found both positive and negative correlations between perfusion and hypoxia in individual tumors, suggesting that tumor biological characteristics may be more reliable indicators of prognosis than perfusion parameters, which can be interpreted in various ways. Regarding MTT, its significantly lower value in the CR group at 3 months can be explained by its inverse relationship with BF, as an increase in BF typically results in a decrease in MTT. Although the increase in MTT at 3 weeks in the CR group was not statistically significant, this trend is consistent with the expected pattern. MTT has been less frequently studied as a predictor of treatment response compared to BF and BV, with only a few studies, including that of Bisdas et al. (22), assessing its predictive value. In the HPV-positive subgroup, significant differences in BF and BV at 3 weeks were observed between the CR and NCR groups. This finding is consistent with the results observed in the overall cohort, which was primarily composed of HPV-positive patients.

DECT QPs evaluation revealed a significantly lower CMA at 3 months in the CR group, with differences in the values of IC and MD at this time point approaching statistical significance (p<0.059 and p<0.056, respectively). These three QPs are related to contrast within the tumor, and their lower values in the CR group suggest reduced tumor tissue involvement at 3 months, reflecting an effective treatment response. Various studies have assessed pre- and post-treatment DECT-derived QPs in patients with HNC who underwent surgery or RT. These studies consistently show higher IC values, reflecting increased tumor perfusion and contrast uptake, to be linked to worse prognosis (23–27). This aligns with our findings, where higher QPs are associated with more aggressive tumor behavior.

When it comes to the HPV status, our observations revealed that, while Nesteruk et al. (28) reported significantly increased BF in HPV-positive HNC tumors, no significant differences were found in the absolute values of perfusion-related QPs between HPV-positive and HPV-negative lesions in our study. However, two delta values Δ CMA (3W-0) and Δ MD (3W-0)—were significantly higher in the HPV-positive group (p<0.043 and p<0.055, respectively). This could be attributed to the generally better response of HPV-positive tumors to RT, which is thought to induce a stronger and more rapid inflammatory response, increasing blood flow into the lesion (28). An alternative hypothesis is that HPV-positive tumors, which are often more cystic than other tumors (29), may have decreased cellularity. This could reduce resistance to BF, potentially explaining the observed but non-significant decrease in MTT in the HPV-positive group (p<0.076). Interestingly, these results were not correlated with other perfusion-related QPs such as BF and BV, which are also influenced by blood perfusion, suggesting that additional mechanisms may be involved in the differences observed in the delta values. The small difference in timing acquisition or post-processing intrinsic weaknesses could also be determinant factors of this uncorrelation.

This study has several strengths. Firstly patient’s tumors were imaged prospectively, allowing for a more robust and reliable dataset. Variables were carefully predetermined and measured by experienced clinicians throughout the study. Also, all assessments described earlier were conducted in a consistent manner. A notable strength is the inclusion of imaging data taken midway through the RT treatment (at 3 weeks), which is a novel approach not commonly used in previous studies. Additionally, this study uniquely explores the potential differences in DECT-derived quantitative parameters between HPV-positive and HPV-negative patients, an area that has not been previously addressed in the literature. However, there are also many limitations to consider. The study’s subgroups were relatively small and asymmetrical, with multiple comparisons conducted and no correction for multiple test applied, which may have influenced the statistical outcomes with the likelihood of obtaining false-positive results, especially in the HPV-stratified subgroups comparisons, where these analyses are underpowered and the corresponding p-values should be interpreted with caution. Also, the fact that, for a few patients (n=2), quantitative parameters were only measured in pathological lymph nodes (see Results section) may also have influenced the final outcomes, although their very small number and their inclusion in the larger CR group likely had little influence on the final results. All ROI being placed by a single observer may have also contributed to intra-operator variations, conducting to potential mistakes. Furthermore, our objective being to assess treatment response using CT imaging alone, evaluation was conducted using the RECIST 1.1 criteria, which primarily focuses on the morphological changes in tumors (30). This approach may not fully capture the effects of treatments like CRT, which may alter tumor content, particularly vascularization, without significantly changing tumor size. Additionally, the presence of large cystic lesions or radionecrosis, often seen in HPV-positive patients (29), could distort the assessment of treatment response. Finally, our study was designed to assess treatment response using QPs from a single ROI, which may create limitations. Recent studies showed that radiomics or texture analysis may provide complementary information on spatial heterogeneity, such as Bogowicz et al. (31), who showed that conventional CT radiomics may predict HPV status and local tumor control in HNC after chemoradiotherapy.

## Conclusion

This exploratory study, conducted within the Swall-PEG trial, suggests potential associations between PCT and DECT-derived quantitative parameters and treatment response in advanced HNC. Notably, PCT measurements at three weeks conducted mid RT may offer an opportunity for early response assessment. However, as this was a secondary endpoint analysis not prospectively designed for biomarker validation, these hypothesis generating findings require confirmation in dedicated prospective studies with larger cohorts before clinical implementation. Future research should also evaluate additional QPs, such as HU for DECT, and establish validated thresholds for clinical decision-making.

## Data Availability

The raw data supporting the conclusions of this article will be made available by the authors, without undue reservation.

## References

[1] Marur S D’Souza G Westra WH Forastiere AA . HPV-associated head and neck cancer: a virus-related cancer epidemic. Lancet Oncol. (2010) 11:781–9. doi: 10.1016/S1470-2045(10)70017-6. PMID: 20451455 PMC5242182

[2] Ang KK Harris J Wheeler R Weber R Rosenthal DI Nguyen-Tân PF . Human papillomavirus and survival of patients with oropharyngeal cancer. N Engl J Med. (2010) 363:24–35. doi: 10.1056/NEJMoa0912217. PMID: 20530316 PMC2943767

[3] Gendreizig S Martínez-Ruiz L López-Rodríguez A Pabla H Hose L Brasch F . Human papillomavirus-associated head and neck squamous cell carcinoma cells lose viability during triggered myocyte lineage differentiation. Cell Death Dis. (2024) 15:1–8. doi: 10.1038/s41419-024-06867-4. PMID: 39030166 PMC11271587

[4] Machiels JP Leemans CR Golusinski W Grau C Licitra L Gregoire V . Squamous cell carcinoma of the oral cavity, larynx, oropharynx and hypopharynx: EHNS–ESMO–ESTRO Clinical Practice Guidelines for diagnosis, treatment and follow-up†. Ann Oncol. (2020) 31:1462–75. doi: 10.1016/j.annonc.2020.07.011. PMID: 33239190

[5] Cooper JS Pajak TF Forastiere AA Jacobs J Campbell BH Saxman SB . Postoperative concurrent radiotherapy and chemotherapy for high-risk squamous-cell carcinoma of the head and neck. N Engl J Med. (2004) 350:1937–44. doi: 10.1056/NEJMoa032646. PMID: 15128893

[6] Bernier J Domenge C Ozsahin M Matuszewska K Lefèbvre JL Greiner RH . Postoperative irradiation with or without concomitant chemotherapy for locally advanced head and neck cancer. N Engl J Med. (2004) 350:1945–52. doi: 10.1056/NEJMoa032641. PMID: 15128894

[7] Vandecaveye V Dirix P De Keyzer F de Beeck KO Vander Poorten V Roebben I . Predictive value of diffusion-weighted magnetic resonance imaging during chemoradiotherapy for head and neck squamous cell carcinoma. Eur Radiol. (2010) 20:1703–14. doi: 10.1007/s00330-010-1734-6. PMID: 20179939

[8] Preda L Calloni SF Moscatelli MEM Cossu Rocca M Bellomi M . Role of CT perfusion in monitoring and prediction of response to therapy of head and neck squamous cell carcinoma. BioMed Res Int. (2014) 2014:917150. doi: 10.1155/2014/917150. PMID: 25140324 PMC4129140

[9] Pietsch C de Galiza Barbosa F Hüllner MW Schmid DT Haerle SK Huber GF . Combined PET/CT-perfusion in patients with head and neck cancers might predict failure after radio-chemotherapy: a proof of concept study. BMC Med Imaging. (2015) 15:60. doi: 10.1186/s12880-015-0102-z. PMID: 26714448 PMC4696250

[10] Chellini D Kinman K . Dual-energy CT principles and applications. Radiol Technol. (2020) 91:561CT–76CT 32606242

[11] Dragan T Van Gossum A Duprez F Lalami Y Lefebvre Y Mootassim-Billah S . Patient-reported outcomes in terms of swallowing and quality of life after prophylactic versus reactive percutaneous endoscopic gastrostomy tube placement in advanced oropharyngeal cancer patients treated with definitive chemo-radiotherapy: Swall PEG study. Trials. (2022) 23:1036. doi: 10.1186/s13063-022-06991-6. PMID: 36539781 PMC9768988

[12] Grégoire V Evans M Le QT Bourhis J Budach V Chen A . Delineation of the primary tumour Clinical Target Volumes (CTV-P) in laryngeal, hypopharyngeal, oropharyngeal and oral cavity squamous cell carcinoma: AIRO, CACA, DAHANCA, EORTC, GEORCC, GORTEC, HKNPCSG, HNCIG, IAG-KHT, LPRHHT, NCIC CTG, NCRI, NRG Oncology, PHNS, SBRT, SOMERA, SRO, SSHNO, TROG consensus guidelines. Radiother Oncol J Eur Soc Ther Radiol Oncol. (2018) 126:3–24. doi: 10.1016/j.radonc.2017.10.016. PMID: 29180076

[13] Brouwer CL Steenbakkers RJHM Bourhis J Budach W Grau C Grégoire V . CT-based delineation of organs at risk in the head and neck region: DAHANCA, EORTC, GORTEC, HKNPCSG, NCIC CTG, NCRI, NRG Oncology and TROG consensus guidelines. Radiother Oncol J Eur Soc Ther Radiol Oncol. (2015) 117:83–90. doi: 10.1016/j.radonc.2015.07.041. PMID: 26277855

[14] Eisenhauer EA Therasse P Bogaerts J Schwartz LH Sargent D Ford R . New response evaluation criteria in solid tumours: revised RECIST guideline (version 1.1). Eur J Cancer Oxf Engl 1990. (2009) 45:228–47. doi: 10.1016/j.ejca.2008.10.026. PMID: 19097774

[15] Hermans R Meijerink M Van den Bogaert W Rijnders A Weltens C Lambin P . Tumor perfusion rate determined noninvasively by dynamic computed tomography predicts outcome in head-and-neck cancer after radiotherapy. Int J Radiat Oncol Biol Phys. (2003) 57:1351–6. doi: 10.1016/s0360-3016(03)00764-8. PMID: 14630273

[16] Truong MT Saito N Ozonoff A Wang J Lee R Qureshi MM . Prediction of locoregional control in head and neck squamous cell carcinoma with serial CT perfusion during radiotherapy. AJNR Am J Neuroradiol. (2011) 32:1195–201. doi: 10.3174/ajnr.A2501. PMID: 21757530 PMC7966050

[17] García-Figueiras R Goh VJ Padhani AR Baleato-González S Garrido M León L . CT perfusion in oncologic imaging: A useful tool? Am J Roentgenol. (2013) 200:8–19. doi: 10.2214/AJR.11.8476. PMID: 23255736

[18] Yoshimura M Itasaka S Harada H Hiraoka M . Microenvironment and radiation therapy. BioMed Res Int. (2013) 2013:685308. doi: 10.1155/2013/685308. PMID: 23509762 PMC3591225

[19] Carmeliet P Jain RK . Molecular mechanisms and clinical applications of angiogenesis. Nature. (2011) 473:298–307. doi: 10.1038/nature10144. PMID: 21593862 PMC4049445

[20] Cater DB Grigson CM Watkinson DA . Changes of oxygen tension in tumours induced by vasoconstrictor and vasodilator drugs. Acta Radiol. (1962) 58:401–34. doi: 10.3109/00016926209169582. PMID: 14019328

[21] Grkovski M Schöder H Lee NY Carlin SD Beattie BJ Riaz N . Multiparametric imaging of tumor hypoxia and perfusion with 18F-fluoromisonidazole dynamic PET in head and neck cancer. J Nucl Med. (2017) 58:1072–80. doi: 10.2967/jnumed.116.188649. PMID: 28183993 PMC5493008

[22] Bisdas S Nguyen SA Anand SK Glavina G Day T Rumboldt Z . Outcome prediction after surgery and chemoradiation of squamous cell carcinoma in the oral cavity, oropharynx, and hypopharynx: use of baseline perfusion CT microcirculatory parameters vs. tumor volume. Int J Radiat Oncol Biol Phys. (2009) 73:1313–8. doi: 10.1016/j.ijrobp.2008.06.1956. PMID: 18963541

[23] Yang L Luo D Yi J Li L Zhao Y Lin M . Therapy effects of advanced hypopharyngeal and laryngeal squamous cell carcinoma: evaluated using dual-energy CT quantitative parameters. Sci Rep. (2018) 8:1. doi: 10.1038/s41598-018-27341-0. PMID: 29899458 PMC5998143

[24] Bahig H Lapointe A Bedwani S Guise J Lambert L Filion E . Dual-energy computed tomography for prediction of loco-regional recurrence after radiotherapy in larynx and hypopharynx squamous cell carcinoma. Eur J Radiol. (2019) 110:1–6. doi: 10.1016/j.ejrad.2018.11.005. PMID: 30599844

[25] Yang X Hu H Zhang F Li D Yang Z Shi G . Preoperative prediction of the aggressiveness of oral tongue squamous cell carcinoma with quantitative parameters from dual-energy computed tomography. Front Oncol. (2022) 12:904471. doi: 10.3389/fonc.2022.904471. PMID: 35814448 PMC9260668

[26] Shen H Huang Y Yuan X Liu D Tu C Wang Y . Using quantitative parameters derived from pretreatment dual-energy computed tomography to predict histopathologic features in head and neck squamous cell carcinoma. Quant Imaging Med Surg. (2022) 12:1243–56. doi: 10.21037/qims-21-650. PMID: 35111620 PMC8739140

[27] Takumi K Hakamada H Nagano H Fukukura Y Kumagae Y Sakai O . Usefulness of dual-layer spectral CT in follow-up examinations: diagnosing recurrent squamous cell carcinomas in the head and neck. Jpn J Radiol. (2021) 39:324–32. doi: 10.1007/s11604-020-01071-8. PMID: 33215300

[28] Nesteruk M Lang S Veit-Haibach P Studer G Stieb S Glatz S . Tumor stage, tumor site and HPV dependent correlation of perfusion CT parameters and [18F]-FDG uptake in head and neck squamous cell carcinoma. Radiother Oncol J Eur Soc Ther Radiol Oncol. (2015) 117:125–31. doi: 10.1016/j.radonc.2015.09.026. PMID: 26452710

[29] Touska P Connor S . Imaging of human papilloma virus associated oropharyngeal squamous cell carcinoma and its impact on diagnosis, prognostication, and response assessment. Br J Radiol. (2022) 95:20220149. doi: 10.1259/bjr.20220149. PMID: 35687667 PMC9815738

[30] Grimaldi S Terroir M Caramella C . Advances in oncological treatment: limitations of RECIST 1.1 criteria. Q J Nucl Med Mol Imaging Off Publ Ital Assoc Nucl Med AIMN Int Assoc Radiopharmacol IAR Sect Soc Of. (2018) 62:129–39. doi: 10.23736/S1824-4785.17.03038-2. PMID: 29166754

[31] Bogowicz M Riesterer O Ikenberg K Stieb S Moch H Studer G . Computed tomography radiomics predicts HPV status and local tumor control after definitive radiochemotherapy in head and neck squamous cell carcinoma. Int J Radiat Oncol Biol Phys. (2017) 99:921–8. doi: 10.1016/j.ijrobp.2017.06.002. PMID: 28807534

