# Supplementary material for: Dual-energy and perfusion CT for predicting response to chemo-radiotherapy in head and neck cancer: an exploratory study
Source: Front Oncol. 2026 May 5;16:1762607. doi: 10.3389/fonc.2026.1762607 (PMC13183546; doi:10.3389/fonc.2026.1762607)
Supplement: Supplementary file 1 [file Table1.docx]

**Appendix A**

Did not attend 3 and 12 months follow-up but other CT in another center at 12 months showed complete response-> reintegrated.

(n = 1)

Excluded: Death before 3 months follow-up

(n = 2)

3 weeks imaging

(n = 33)

Did not undergo 3 weeks follow-up but attended later the 3 months one

(n = 2)

Excluded (n = 1): death before beginning of imaging

Screen-failure (n = 2)

Excluded (n = 1): did not meet inclusion criteria

Eligible subjects from the Swall- PEG clinical trial

(n = 39)

**Figure A :** Patient’s Diagram flow

12 months imaging

(n = 33)

3 months imaging (n=32)

Pre-treatment imaging (baseline) (n = 35)

Enrolled (n = 36)

|  | **Complete response (CR)** | **Partial Response (PR)** | **Stable disease (SD)** | **Progressive disease (PD)** |
| --- | --- | --- | --- | --- |
| 3 weeks (*n* = 33) | 4 | 9 | 19 | 1 |
|  |  |  |  |  |
| 3 months (*n* = 32) | 18 | 14 | 0 | 0 |
|  |  |  |  |  |
| 12 months (*n* = 33) | 26 | 3 | 0 | 4 |
|  |  |  |  |  |

**Table A:** Patients’ RECIST 1.1 assessment analyzed at each period.

| **Variable** |  | **CR**, n = 26^1^ |  | **NCR**, n = 8^1*^ |  | **p-value**^2^ |
| --- | --- | --- | --- | --- | --- | --- |
| Sex |  |  |  |  |  | 0.6 |
| - Male |  | 21 (81%) |  | 5 (71%) |  |  |
| - Female |  | 5 (19%) |  | 2 (29%) |  |  |
| Tabacco use |  |  |  |  |  | 0.11 |
| - Never |  | 6 (23%) |  | 1 (14%) |  |  |
| - Active + Former |  | 20 (77%) |  | 6 (86%) |  |  |
| - Pack-years |  | 27 (range 11 - 38) |  | 34 (range 30 - 52) |  | 0.079 |
| Alcohol consumption |  |  |  |  |  | 0.4 |
| - Never |  | 4 (15%) |  | 1 (14%) |  |  |
| - Active + Former |  | 22 (85%) |  | 6 (86%) |  |  |
| BMI |  | 25.6 (range 21.5 - 28.5) |  | 24.4 (range 20.0 - 25.9) |  | 0.3 |
| Oropharynx HPV |  |  |  |  |  | 0.2 |
| - Pos |  | 18 (82%) |  | 3 (50%) |  |  |
| - Neg |  | 4 (18%) |  | 3 (50%) |  |  |
| Age |  | 64 (range 58 - 68) |  | 66 (range 63 - 70) |  | 0.5 |
| Chemotherapy |  |  |  |  |  | >0.9 |
| - Regimen 1 |  | 13 (50%) |  | 3 (43%) |  |  |
| - Regimen 2 |  | 13 (50%) |  | 4 (57%) |  |  |
| ^1^ n (%)- Median + interquartile range (IQR)  ² Fisher’s exact test- Wilcoxon rank sum exact test- Wilcoxon rank sum test | | | | | | |

*7 patients in NCR group but 8 tumors analyzed due to development of additional tumor in one of the patients (cf. results)

**Table B**: Multivariate analysis between complete response (CR) and non-complete (NCR) groups

| **Variable** |  | **HPV-pos (range)**, n = 21 | |  | **HPV-neg (range)**, n = 7 | |  | **p-value**^2^ |
| --- | --- | --- | --- | --- | --- | --- | --- | --- |
|  |  | **n** | **Summary**^1^ |  | **n** | **Summary**^1^ |  |  |
| GTVp |  | 21 | 9.18 (6.89 -20.47) |  | 7 | 18.69 (7.14 - 28.15) |  | 0.18 |
| GTVn |  | 20 | 16.22 (7.5 -39.7) |  | 6 | 10.82 (8.65 - 58.54) |  | 0.67 |
| PCT absolute values |  |  |  |  |  |  |  |  |
| - BF_0 |  | 21 | 102 (84 -122) |  | 7 | 103 (77 - 115) |  | >0.9 |
| - BF_3W |  | 18 | 131 (76 - 184) |  | 7 | 87 (78 - 114) |  | 0.4 |
| - BF_3M |  | 4 | 55 (52 - 63) |  | 3 | 86 (71 - 141) |  | 0.3 |
| - BV_0 |  | 21 | 8.39 (7.04 - 10.76) |  | 7 | 6.93 (6.65 - 8.78) |  | 0.3 |
| - BV_3W |  | 18 | 10.7 (9.1 - 13.8) |  | 7 | 9.3 (7.8 - 12.4) |  | 0.8 |
| - BV_3M |  | 4 | 5.7 (4.7 - 6.2) |  | 3 | 10.4 (7.1 - 14.2) |  | 0.2 |
| - MTT_0 |  | 21 | 5.68 (4.90 - 6.18) |  | 7 | 5.06 (4.66 - 6.32) |  | 0.7 |
| - MTT_3W |  | 18 | 5.47 (4.98 - 5.95) |  | 7 | 6.48 (5.70 - 6.84) |  | 0.076 |
| - MTT_3M |  | 4 | 6.37 (6.33 - 6.39) |  | 3 | 6.56 (5.69 - 6.69) |  | 0.7 |
| DECT absolute values |  |  |  |  |  |  |  |  |
| - MD_0 |  | 21 | 117 (104 - 133) |  | 7 | 132 (110 - 146) |  | 0.4 |
| - MD_3W |  | 18 | 160 (140 - 181) |  | 7 | 139 (138 - 168) |  | 0.2 |
| - MD_3M |  | 4 | 119 (74 - 130) |  | 3 | 129 (107 - 164) |  | 0.4 |
| - VNCA_0 |  | 21 | 42 (37 - 50) |  | 7 | 37 (31 - 43) |  | 0.14 |
| - VNCA_3W |  | 18 | 35 (30 - 41) |  | 7 | 31 (28 - 33) |  | 0.2 |
| - VNCA_3M |  | 4 | 33 (21 - 34) |  | 3 | 22 (14 - 27) |  | 0.3 |
| - CMA_0 |  | 21 | 77 (61 - 99) |  | 7 | 93 (75 - 118) |  | 0.13 |
| - CMA_3W |  | 18 | 128 (101 - 148) |  | 7 | 109 (99 - 138) |  | 0.4 |
| - CMA_3M |  | 4 | 93 (86 - 113) |  | 3 | 129 (82 - 137) |  | 0.8 |
| - IC_0 |  | 21 | 2.30 (1.95 - 3.18) |  | 7 | 2.90 (2.35 - 3.63) |  | 0.14 |
| - IC_3W |  | 18 | 4.05 (3.10 - 4.75) |  | 7 | 3.60 (3.40 - 4.40) |  | 0.9 |
| - IC_3M |  | 4 | 3.60 (1.40 - 3.60) |  | 3 | 4.30 (2.90 - 4.50) |  | 0.8 |
| - FFV_0 |  | 21 | 10.5 (5.5 - 14.1) |  | 7 | 13.5 (10.3 - 17.1) |  | 0.2 |
| - FFV_3W |  | 18 | 15 (11 - 23) |  | 7 | 17 (16 - 22) |  | 0.2 |
| - FFV_3M |  | 4 | 18 (17 - 60) |  | 3 | 24 (21 - 37) |  | 0.7 |
| PCT Δ (t1-0) |  |  |  |  |  |  |  |  |
| - Δ BF (3W-0) |  | 18 | 34.12 (-22.04 -65.2) |  | 7 | 13.12 (-9.32 - 73.77) |  | 0.41 |
| - Δ BF (3M-0) |  | 5 | -28.31 (-30.01 - 5.18) |  | 2 | -12.61 (-57.29 - 32.07) |  | 0.5 |
| - Δ BV (3W-0) |  | 18 | 2.28 (-0.72 - 4.76) |  | 7 | 3.29 (0.72 - 5.35) |  | 0.33 |
| - Δ MTT (3W-0) |  | 18 | -0.09 (-0.64 - 0.94) |  | 7 | 0.39 (-0.48 - 2.27) |  | 0.17 |
| - DECT Δ (t1-0) |  |  |  |  |  |  |  |  |
| - Δ MD (3W-0) |  | 18 | 35.35 (15.7 - 51) |  | 7 | 18.8 (-22.5 - 33.7) |  | 0.055 |
| - Δ CMA (3W-0) |  | 18 | 41.7 (25.4 - 51.8) |  | 7 | 22.8 (17.8 - 32.7) |  | **0.043** |
| - Δ IC (3W-0) |  | 18 | 1.25 (0.7 - 1.7) |  | 7 | 1 (0.8 - 1) |  | 0.12 |
| ^1^Median + interquartile range (IQR)  ^2^Wilcoxon rank sum exact test- Wilcoxon rank sum test | | | | | | | | |

**Table C:** Comparison of perfusion and dual-energy computed tomography quantitative parameters between HPV-positive and negative groups in patients with oropharyngeal tumor***:*** ***BF:*** *Blood Flow;* ***BV:*** *Blood Volume;* ***MTT*** *:Mean Transit Time;* ***VNCA:*** *Virtual non contrast attenuation;* ***CMA:*** *Contrast media attenuation;* ***MD:*** *Mixed density;* ***IC:*** *Iodine concentration;* ***FFV****: Fat fraction Volume;* ***GTVp****: Gross tumor volume,* ***GTVn****: Gross nodal volume;;****Δ BF (0-3W)****: Difference in Blood Flow between 3 weeks and baseline);* ***Δ BF (0-3M)****: Difference in Blood Flow between 3 months and baseline;* ***Δ BV (0-3W)****: Difference in Blood Volume between 3 weeks and baseline;* ***Δ BV (0-3M)****: Difference in Blood Volume between 3 months and baseline;* ***Δ IC (0-3W)****: Difference in Iodine Concentration between 3 weeks and baseline;* ***Δ IC (0-3M)****: Difference in Iodine Concentration between 3 months and baseline;* ***Δ CMA (0-3W)****: Difference in Contrast Media Attenuation between 3 weeks and baseline;* ***Δ CMA (3M-0)****: Difference in Contrast Media Attenuation between 3 months and baseline;* ***Δ MD (0-3W)****: Difference in Mixed Density between 3 weeks and baseline;* ***Δ MD (3M-0)****: Difference in Mixed Density between 3 months and baseline;* ***Δ MTT (0-3W)****: Difference in Mean Transit Time between 3 weeks and baseline;* ***Δ MTT (3M-0)****: Difference in Mean Transit Time between 3 months and baseline.*

| **Variable** |  | **CR (range)**- n = 4 | |  | **NCR (range)**- n = 3 | |  | **p-value**^2^ |
| --- | --- | --- | --- | --- | --- | --- | --- | --- |
|  |  | **n** | **Summary**^1^ |  | **n** | **Summary**^1^ |  |  |
| PCT absolute values |  |  |  |  |  |  |  |  |
| - BF_0 |  | 4 | 103 (81 - 115) |  | 3 | 96 (78 - 122) |  | >0.9 |
| - BF_3W |  | 4 | 100 (70 - 172) |  | 3 | 87 (82 - 92) |  | >0.9 |
| - BV_0 |  | 4 | 7.49 (6.89 - 8.78) |  | 3 | 6.67 (6.27 - 7.91) |  | 0.5 |
| - BV_3W |  | 4 | 10.8 (7.2 - 14.8) |  | 3 | 8.5 (8.1 - 10.0) |  | 0.7 |
| - MTT_0 |  | 4 | 5.48 (4.98 - 6.33) |  | 3 | 4.82 (4.50 - 5.42) |  | 0.6 |
| - MTT_3W |  | 4 | 6.31 (5.40 - 6.73) |  | 3 | 6.84 (6.27 - 7.02) |  | 0.4 |
| DECT absolute values |  |  |  |  |  |  |  |  |
| - MD_0 |  | 4 | 132 (115 - 156) |  | 3 | 123 (110 - 138) |  | 0.8 |
| - MD_3W |  | 4 | 139 (124 - 161) |  | 3 | 140 (139 - 167) |  | 0.5 |
| - VNCA_0 |  | 4 | 39 (35 - 43) |  | 3 | 34 (25 - 39) |  | 0.5 |
| - VNCA_3W |  | 4 | 31 (28 - 37) |  | 3 | 32 (30 - 32) |  | >0.9 |
| - CMA_0 |  | 4 | 97 (71 - 118) |  | 3 | 93 (88 - 101) |  | >0.9 |
| - CMA_3W |  | 4 | 104 (85 - 130) |  | 3 | 110 (108 - 137) |  | 0.4 |
| - IC_0 |  | 4 | 2.95 (2.28 - 3.63) |  | 3 | 2.90 (2.68 - 3.18) |  | >0.9 |
| - IC_3W |  | 4 | 3.60 (3.40 - 4.25) |  | 3 | 3.60 (3.45 - 4.30) |  | 0.9 |
| - FFV_0 |  | 4 | 13.5 (10.1 - 14.8) |  | 3 | 15.0 (11.4 - 21.2) |  | 0.6 |
| - FFV_3W |  | 4 | 18.7 (17.1 - 24.5) |  | 3 | 15.9 (15.7 - 18.8) |  | 0.5 |
| ^1^Median + interquartile range (IQR)  ^2^Wilcoxon rank sum exact test- Wilcoxon rank sum test | | | | | | | | |

**Table D:** Comparison of perfusion and dual-energy computed tomography quantitative parameters measured at baseline (0), 3 weeks (3W) and 3 months (3M) post beginning of treatment between complete and non-complete response cohorts in HPV-negative patients: ***CR****: complete response;* ***NCR****: non-complete response* ***BF:*** *Blood Flow* ***BV****: Blood Volume;* ***MTT****: Mean Transit Time;* ***VNCA****: Virtual non contrast attenuation;* ***CMA****: Contrast media attenuation;* ***MD****: Mixed density;* ***IC****: Iodine concentration;* ***FFV****: Fat fraction Volume.*

| **Variable** |  | **CR (range)**, n = 17^1^ | |  | **NCR (range)**, n = 3^1^ | |  | **p-value**^2^ |
| --- | --- | --- | --- | --- | --- | --- | --- | --- |
|  |  | **n** | **Summary** |  | **n** | **Summary** |  |  |
| PCT absolute values |  |  |  |  |  |  |  |  |
| - BF_0 |  | 17 | 104 (91 - 127) |  | 3 | 85 (61 - 93) |  | 0.15 |
| - BF_3W |  | 15 | 143 (113 - 192) |  | 3 | 60 (51 - 61) |  | **0.017** |
| - BF_3M |  | 3 | 63 (57 - 80) |  | 2 | 54 (53 - 54) |  | 0.8 |
| - BV_0 |  | 17 | 8.80 (7.29 - 10.78) |  | 3 | 6.64 (5.26 - 7.42) |  | 0.11 |
| - BV_3W |  | 15 | 11.9 (9.8 - 14.3) |  | 3 | 4.8 (4.5 - 5.0) |  | **0.017** |
| - BV_3M |  | 3 | 6.16 (5.91 - 7.64) |  | 2 | 4.26 (4.02 - 4.49) |  | 0.2 |
| - MTT_0 |  | 17 | 5.65 (4.58 - 6.19) |  | 3 | 5.70 (5.52 - 5.73) |  | >0.9 |
| - MTT_3W |  | 15 | 5.32 (4.80 - 5.85) |  | 3 | 5.51 (5.49 - 6.31) |  | 0.3 |
| - MTT_3M |  | 3 | 6.39 (6.38 - 6.51) |  | 2 | 5.53 (5.13 - 5.93) |  | 0.2 |
| DECT absolute values |  |  |  |  |  |  |  |  |
| - MD_0 |  | 17 | 117 (103 - 132) |  | 3 | 117 (111 - 145) |  | 0.5 |
| - MD_3W |  | 15 | 167 (140 - 179) |  | 3 | 142 (141 - 165) |  | >0.9 |
| - MD_3M |  | 3 | 74 (71 - 111) |  | 2 | 124 (122 - 127) |  | 0.8 |
| - VNCA_0 |  | 17 | 40 (35 - 48) |  | 3 | 50 (46 - 57) |  | 0.14 |
| - VNCA_3W |  | 15 | 35 (28 - 41) |  | 3 | 39 (36 - 40) |  | 0.8 |
| - VNCA_3M |  | 3 | 34 (24 - 41) |  | 2 | 27 (24 - 30) |  | 0.8 |
| - CMA_0 |  | 17 | 78 (63 - 99) |  | 3 | 75 (65 - 90) |  | >0.9 |
| - CMA_3W |  | 15 | 130 (101 - 147) |  | 3 | 101 (101 - 128) |  | >0.9 |
| - CMA_3M |  | 3 | 93 (58 - 103) |  | 2 | 117 (102 - 133) |  | 0.8 |
| - IC_0 |  | 17 | 2.30 (2.00 - 3.10) |  | 3 | 2.30 (2.00 - 3.05) |  | >0.9 |
| - IC_3W |  | 15 | 4.10 (3.10 - 4.70) |  | 3 | 3.10 (3.10 - 4.30) |  | >0.9 |
| - IC_3M |  | 3 | 3.60 (2.10 - 3.60) |  | 2 | 3.30 (2.35 - 4.25) |  | 0.8 |
| - FFV_0 |  | 17 | 12.2 (8.9 - 14.2) |  | 3 | 5.5 (5.0 - 7.7) |  | 0.2 |
| - FFV_3W |  | 15 | 16 (11 - 22) |  | 3 | 11 (11 - 20) |  | >0.9 |
| - FFV_3M |  | 3 | 18 (13 - 40) |  | 2 | 39 (28 - 49) |  | >0.9 |
| ^1^Median + interquartile range (IQR)  ^2^Wilcoxon rank sum exact test- Wilcoxon rank sum test | | | | | | | | |

**Table E:** Comparison of perfusion and dual-energy computed tomography quantitative parameters measured at baseline (0), 3 weeks (3W) and 3 months (3M) post beginning of treatment between complete and non-complete response cohorts in HPV-positive patients: ***CR****: complete response;* ***NCR****: non-complete response* ***BF:*** *Blood Flow* ***BV****: Blood Volume;* ***MTT****: Mean Transit Time;* ***VNCA****: Virtual non contrast attenuation;* ***CMA****: Contrast media attenuation;* ***MD****: Mixed density;* ***IC****: Iodine concentration;* ***FFV****: Fat fraction Volume.*
